# Fecal Immunoglobulin A Against a Sporozoite Antigen at 12 Months Is Associated With Delayed Time to Subsequent Cryptosporidiosis in Urban Bangladesh: A Prospective Cohort Study

**DOI:** 10.1093/cid/ciz430

**Published:** 2019-05-25

**Authors:** Kevin L Steiner, Mamun Kabir, Jeffrey W Priest, Biplob Hossain, Carol A Gilchrist, Heather Cook, Jennie Z Ma, Poonum S Korpe, Tahmeed Ahmed, A S G Faruque, Rashidul Haque, William A Petri

**Affiliations:** 1 Division of Infectious Diseases and International Health, Department of Medicine, University of Virginia, Charlottesville; 2 icddr,b, Dhaka, Bangladesh; 3 Division of Foodborne, Waterborne and Environmental Diseases, National Center for Emerging and Zoonotic Infectious Diseases, Centers for Disease Control and Prevention, Atlanta, Georgia; 4 Department of Statistics, School of Medicine, University of Virginia, Charlottesville; 5 Division of Biostatistics, Department of Public Health Sciences, School of Medicine, University of Virginia, Charlottesville; 6 Department of Epidemiology, Johns Hopkins Bloomberg School of Public Health, Baltimore, Maryland

**Keywords:** cryptosporidiosis, mucosal, fecal, IgA, Cp23

## Abstract

In this prospective cohort study of Bangladeshi children, greater fecal immunoglobulin A, but not plasma immunoglobulin G, directed against the *Cryptosporidium* sporozoite-expressed antigen Cp23 at 12 months of age was associated with delayed time to subsequent cryptosporidiosis. This finding suggests a protective role for mucosal antibody-mediated immunity in naturally exposed children.

Cryptosporidiosis, caused by an intracellular protozoan parasite, is a leading cause of childhood diarrheal morbidity and mortality [1]. Moreover, several recent cohort studies have demonstrated that not only diarrheal, but also subclinical cryptosporidiosis has an adverse effect on growth and development in young children [2, 3]. While development of new treatments for cryptosporidial diarrhea remains an important priority, the detrimental effect of subclinical infection supports the role for development of a vaccine to reduce the full burden of the disease.

While cryptosporidiosis is common among children between 6 months and 2 years of age in many parts of the developing world, the incidence declines rapidly as children age, suggesting a role for adaptive immunity [4]. It is clear that cell-mediated, predominantly interferon gamma (IFN-γ)–driven responses are a critical aspect of an effective adaptive immune response as children age; however, the role of antibody-mediated responses in humans is less understood [5]. We have previously shown that protection from infection during the first year of life is associated with greater anticryptosporidial secretory immunoglobulin A (IgA) in maternal breast milk, supporting a potential role for antibody-mediated mucosal immunity [6]. Cp23 is a sporozoite-expressed protein shed during gliding motility that is highly immunogenic and is capable of inducing detectable serum immunoglobulin G (IgG), IgA, and immunoglobulin M after natural exposure [5, 7, 8]. A human challenge study suggested that the presence of preexisting anti-Cp23 antibodies in blood was associated with less oocyst shedding and fewer symptoms; however, a recent birth cohort study suggested no association of blood IgG with protection [9, 10]. No study to date has assessed fecal anti-Cp23 IgA antibody with longitudinal subsequent follow-up in a naturally exposed population. Here we leverage a well-characterized prospective birth cohort study to investigate whether greater fecal IgA at 1 year of age directed against an immunodominant sporozoite-expressed antigen (Cp23) protects infants from subsequent cryptosporidiosis.

## MATERIALS AND METHODS

The subjects studied were part of an ongoing, prospective birth cohort study established in the urban neighborhood of Mirpur, in Dhaka, Bangladesh [2]. Infants were enrolled and prospectively followed as previously described, including study population, enrollment, surveillance, and polymerase chain reaction (PCR) detection of cryptosporidiosis. Stool was collected monthly and during episodes of diarrhea (≥ 3 loose stools in 24 hours) and tested by real-time PCR for *Cryptosporidium*, as previously described [2]. Plasma was obtained from children at 12 months of age.

Cp23 lacking the GST expression tag was prepared as previously described [11]. Anti-Cp23 plasma IgG was measured using enzyme-linked immunosorbent assay (ELISA) as previously described [11]. Anti-Cp23–specific fecal IgA was measured in the surveillance stool sample obtained at 12 months of age by ELISA adapted from previously described methods [12, 13]. In brief, a standard quantity of cryopreserved stool was thawed, suspended in phosphate-buffered saline (PBS) + ethylenediaminetetraacetic acid + protease inhibitor solution and centrifuged with the supernatant removed and preserved in phenylmethylsulfonyl fluoride and sodium azide. Fecal supernatant was then diluted 10-fold in 0.05% PBS-Tween 20 (PBST) and incubated for 2 hours at room temperature on Cp23-coated microtiter plates. Following incubation and washing with PBST, sequential incubations with biotinylated goat antihuman IgA (Invitrogen) followed by streptavidin-alkaline phosphatase (Invitrogen) were performed. The reaction was developed for 30 minutes with *p*-nitrophenylphosphate (Thermo Scientific) and absorbance values read at 405 nm. Subjects were then divided into the top and bottom 50th percentiles for quantity of fecal anti-Cp23 IgA.

Survival probabilities for time to the first *Cryptosporidium* PCR-positive episode within the second year of life were estimated with the Kaplan-Meier method for anti-Cp23 plasma IgG and fecal IgA as categorical variables of the upper and lower 50th percentiles. Multivariable Cox regression was performed with adjustment of demographic, socioeconomic, and anthropometric covariates. Analyses were performed using R version 3.5.1 with package “survival” version 2.42–3 with function “coxph.”

The Ethics and Research Review Committee at icddr,b approved this study; the Institutional Review Board of the University of Virginia provided a reliance agreement. Informed written consent was obtained from a parent or guardian of each child. Centers for Disease Control and Prevention personnel had no contact with study participants and had no access to identifying information.

## RESULTS

Infants were enrolled at birth between July 2014 and April 2016 and prospectively followed. All 442 children for whom a stool sample was available for IgA testing at 12 months of age were included in the study; 201 were male (45.5%). Average household size was 5.5 and monthly household income was approximately 17 000 Bangladeshi taka (equivalent to 200 US dollars). Average duration of exclusive breastfeeding was 107 days (standard deviation, 71 days). Mean length-for-age *z* score (LAZ) at 12 months was –1.26, and 31% had a LAZ < –2. Ninety-eight percent used municipal water supply and 75% treated the water prior to consumption. Sixty children (13.5%) had detectable cryptosporidiosis only in the first year of life, 173 (39.1%) only in the second year, 66 (14.9%) in both years, and 143 (32.3%) in neither year.

**Figure 1.**
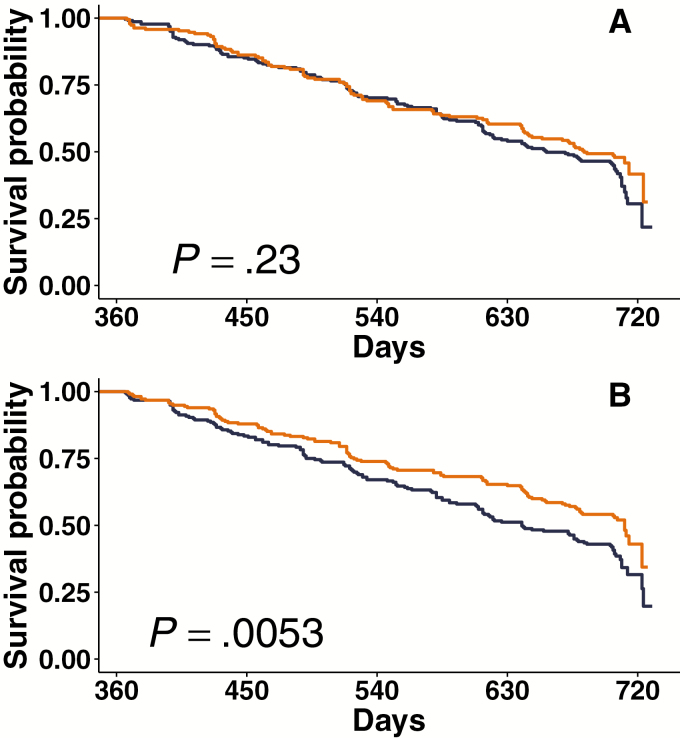
Kaplan-Meier curve showing probability of survival free of *Cryptosporidium* species during the second year of life among infants (n = 442), stratified by amount of anti-Cp23 immunoglobulin. *A*, No difference in subsequent cryptosporidiosis-free survival between children in the upper 50th (orange) and lower 50th percentile (blue) of plasma anti-Cp23 immunoglobulin G measured at 1 year of life. *B*, Children in the upper 50th percentile (orange) of fecal anti-Cp23 immunoglobulin A measured at 1 year of life had a statistically significantly longer subsequent cryptosporidiosis-free survival compared to children in the lower 50th percentile (blue). The y-axis represents the survival probability free from infection, and the x-axis is survival time in days, from 1 to 2 years of life. *P* values by log-rank test.

There was no difference observed in cryptosporidiosis-free survival during the second year of life between children in the upper vs lower 50th percentiles of plasma anti-Cp23 IgG measured at 12 months (Figure 1A). In contrast, children in the upper 50th percentile of fecal anti-Cp23 IgA measured at 12 months had a significantly greater probability of subsequent cryptosporidiosis-free survival compared to children in the lower 50th percentile (Figure 1B; *P* = .0053).

In multivariable Cox regression analysis of time to subsequent cryptosporidiosis, which included demographic, socioeconomic, and infant anthropometric variables, Cp23 IgA in the upper 50th percentile was associated with statistically significant reduction in hazard ratio of 24% (95% confidence interval, .5%–42.3%; *P* = .046). No other analyzed variables achieved statistical significance (Supplementary Table).

## DISCUSSION

In this study, we leveraged a well-characterized, prospective, birth cohort study established in urban Dhaka, Bangladesh, to investigate the role of antibody-mediated immune responses in immunity to cryptosporidiosis. We showed that a greater quantity of fecal IgA at 12 months of age directed against Cp23, a well-described immunodominant antigen expressed on *Cryptosporidium* sporozoites, was associated with a significantly delayed time to subsequent cryptosporidiosis. This association of fecal anti-Cp23 with subsequent protection remained significant in multivariable analysis including other pertinent covariables. In contrast, there was no association of anti-Cp23 IgG measured in plasma with subsequent protection, consistent with a prior birth cohort study in India but differing from an adult challenge model in which preexisting IgG was associated with fewer symptoms and less oocyst shedding [9, 10]. We previously showed a similar association of protection with pathogen-specific fecal IgA but not plasma IgG for *Entamoeba histolytica* [12]. The association of PCR-detected cryptosporidiosis during the first year of life and subsequent delayed time to cryptosporidiosis did not reach statistical significance (data not shown); however, as detected infection during the first year of life occurred in less than one-third of children, power may be insufficient.

Adaptive immune responses are imperative for resolution of cryptosporidiosis and subsequent development of protective immunity [5]. Cell-mediated responses, especially interferon gamma (IFN-γ) production by CD4^+^ T cells, are well described in individuals with prior cryptosporidiosis [14, 15]. The role of antibody responses in cryptosporidiosis is less well defined [5]. The data presented here further support a role for mucosal antibody-mediated immune responses in human disease, consistent with that shown in a previous cohort in which greater anti-*Cryptosporidium* IgA in maternal breast milk was associated with delayed time to initial cryptosporidiosis [6]. A model in which infectious sporozoites are neutralized or impeded from reaching intestinal epithelial cells (IECs) by secreted IgA is a plausible mechanism by which mucosal antibody-mediated immune responses may confer protection. Cell-mediated immunity, including IFN-γ responses, may then be critical for eradication once sporozoites have successfully infected IEC.

Strengths of this study include its size and the prospective cohort design in a setting of naturally occurring exposure to cryptosporidiosis. The use of Cp23, an immunodominant, sporozoite-expressed, vaccine candidate antigen, is an additional strength. Limitations include analysis of anti-Cp23 fecal IgA at a single time point (12 months) and that median IgA was used for stratification for simplicity of analysis, as this study was not designed to establish a particular cut-point as a correlate of protection. Additional studies of the dynamics of fecal anti-Cp23 over time in individual children may provide further insight. We cannot exclude that the measured fecal anti-Cp23 IgA for some children was derived from maternal breast milk; however, mean exclusive breastfeeding in this cohort was approximately 3.5 months and exclusive breastfeeding days was not significant in multivariable analysis, suggesting that this is not likely a major contributor. As surveillance for subclinical cryptosporidiosis occurred monthly, some intervening episodes of nondiarrheal cryptosporidiosis may have been missed. We did not test for cell-mediated immune responses at 12 months as this study intended to focus on antibody-mediated immunity.

These findings further challenge the prevailing paradigm in which cell-mediated, predominantly IFN-γ responses are principal drivers of cryptosporidial immunity and suggest that mucosal antibody-mediated immunity also has an important role. Given the continued significant global burden of cryptosporidiosis on childhood growth and development, these results should further inform vaccine development strategies, which ideally would effectively induce both cell-mediated and mucosal antibody responses and support further consideration of Cp23 as a potential vaccine candidate.

## Supplementary Data

Supplementary materials are available at *Clinical Infectious Diseases* online. Consisting of data provided by the authors to benefit the reader, the posted materials are not copyedited and are the sole responsibility of the authors, so questions or comments should be addressed to the corresponding author.

ciz430_suppl_Supplementary_Table

## Notes

***Author contributions.*** W. A. P., R. H., and A. S. G. F. conceived the study; fieldwork, data gathering, and laboratory studies at icddr,b were performed by B. H., M. A., M. K., and F. T. with supervision by C. A. G., T. A., R. H., and A. S. G. F.; J. W. P. supplied Cp23 and participated in critical review of data; K. L. S., H. C., and J. Z. M. performed statistical analyses; K. L. S. wrote the manuscript with input from C. A. G., J. W. P., P. S. K., H. C., J. Z. M., R. H., and W. A. P. All authors contributed to revisions and approved the final version.

***Acknowledgments.*** The authors express their deepest appreciation to the children and parents from Mirpur for participating in this study.

***Financial support.*** This work was supported by the National Institutes of Health (grant numbers R01 AI043596 to W. A. P. and T32 AI055432 to K. L. S.) and the Bill & Melinda Gates Foundation (grant number OPP1100514 to A. S. G. F.).

***Potential conflicts of interest.*** W. A. P. is a consultant for TechLab, Inc, a company that makes diagnostics for cryptosporidiosis. All other authors report no potential conflicts of interest. All authors have submitted the ICMJE Form for Disclosure of Potential Conflicts of Interest. Conflicts that the editors consider relevant to the content of the manuscript have been disclosed.
