# Supplementary material for: Fecal Immunoglobulin A Against a Sporozoite Antigen at 12 Months Is Associated With Delayed Time to Subsequent Cryptosporidiosis in Urban Bangladesh: A Prospective Cohort Study
Source: Clin Infect Dis. 2019 May 25;70(2):323–6. doi: 10.1093/cid/ciz430 (PMC6938969; doi:10.1093/cid/ciz430)
Supplement: ciz430_suppl_Supplementary_Table [file ciz430_suppl_supplementary_table.docx]

**Supplemental table:** Multivariable Cox regression including demographic, socioeconomic, and anthropometric covariates indicating statistically significant decrease in hazard ration of subsequent cryptosporidiosis for children in the upper 50^th^ percentile of fecal anti-Cp23 IgA.

| **Variable** | **Hazard Ratio** | **95% CI** | **P-value** |
| --- | --- | --- | --- |
| Fecal anti-Cp23 IgA in upper 50^th^ percentile | 0.7576 | 0.5770-0.9947 | 0.046 |
| Sex (female reference) | 1.0803 | 0.8312-1.4039 | 0.564 |
| Income/1000 | 0.9962 | 0.9845-1.0080 | 0.530 |
| Mother education | 1.1368 | 0.8141-1.5874 | 0.452 |
| Mother BMI | 0.9878 | 0.9516-1.0254 | 0.520 |
| Mother age | 0.9867 | 0.9571-1.0172 | 0.390 |
| Household size | 0.9806 | 0.9271-1.0585 | 0.781 |
| Gestational age | 1.0202 | 0.9397-1.1075 | 0.634 |
| HAZ at 12 months | 0.9895 | 0.8617-1.1362 | 0.881 |
| Exclusive breastfeeding days | 0.9988 | 0.9970-1.0007 | 0.214 |
